# *Cyclospora cayetanensis* and Cyclosporiasis: An Update

**DOI:** 10.3390/microorganisms7090317

**Published:** 2019-09-04

**Authors:** Sonia Almeria, Hediye N. Cinar, Jitender P. Dubey

**Affiliations:** 1 Department of Health and Human Services, Food and Drug Administration, Center for Food Safety and Nutrition (CFSAN), Office of Applied Research and Safety Assessment (OARSA), Division of Virulence Assessment, Laurel, MD 20708, USA; 2 Animal Parasitic Disease Laboratory, United States Department of Agriculture, Agricultural Research Service, Beltsville Agricultural Research Center, Building 1001, BARC-East, Beltsville, MD 20705-2350, USA

**Keywords:** Cyclospora, humans, epidemiology, diagnosis, life cycle, control

## Abstract

*Cyclospora cayetanensis* is a coccidian parasite of humans, with a direct fecal–oral transmission cycle. It is globally distributed and an important cause of foodborne outbreaks of enteric disease in many developed countries, mostly associated with the consumption of contaminated fresh produce. Because oocysts are excreted unsporulated and need to sporulate in the environment, direct person-to-person transmission is unlikely. Infection by *C. cayetanensis* is remarkably seasonal worldwide, although it varies by geographical regions. Most susceptible populations are children, foreigners, and immunocompromised patients in endemic countries, while in industrialized countries, *C. cayetanensis* affects people of any age. The risk of infection in developed countries is associated with travel to endemic areas and the domestic consumption of contaminated food, mainly fresh produce imported from endemic regions. Water and soil contaminated with fecal matter may act as a vehicle of transmission for *C. cayetanensis* infection. The disease is self-limiting in most immunocompetent patients, but it may present as a severe, protracted or chronic diarrhea in some cases, and may colonize extra-intestinal organs in immunocompromised patients. Trimetoprim-sulfamethoxazole is the antibiotic of choice for the treatment of cyclosporiasis, but relapses may occur. Further research is needed to understand many unknown epidemiological aspects of this parasitic disease. Here, we summarize the biology, epidemiology, outbreaks, clinical symptoms, diagnosis, treatment, control and prevention of *C. cayetanensis*; additionally, we outline future research needs for this parasite.

## 1. Introduction

*Cyclospora* spp. are protozoan parasites in the phylum Apicomplexa, class Sporozoasida, subclass Coccidiasina, order Eimeriorina, family Eimeriidae. *Cyclospora cayetanensis* is the only species of the genus *Cyclospora* known to infect humans. *C. cayetanensis* was first described and named 25 years ago [1,2]. Earlier, there were reports of organisms initially known as *Cyanobacterium*-like body or coccidian-like body, blue-green alga, large *Cryptosporidium* or small *Isospora*-like organisms associated with diarrhea in humans, which in retrospect were probably *C. cayetanensis* [3].

Some other species of the genus *Cyclospora* have been described in non-human primates [4,5,6,7,8,9] and most probably are host-specific. *Cyclospora macacae* is the most recently described species, reported from rhesus monkeys (*Macaca mulatta*) [10]. *Cyclospora* oocysts were reported in chimpanzees (*Pan troglodytes*) and Cynomolgus monkeys (*Macaca fascicularis*) with similar sequences to those determined in *C. cayetanensis*, based on ITS-2 [11]. There are still questions regarding the host specificity of these parasites and whether they are transmissible to humans; caution is needed in assigning species status [11]. Attempts to infect numerous animals with *C. cayetanensis* have been unsuccessful, suggesting host specificity [12] and it is assumed that all human infections are caused by *C. cayetanensis*. Oocysts resembling *Cyclospora* have been found in the feces of several animals, including dogs, mice, rats, monkeys, ducks, chickens, and other avian species [13,14,15,16,17,18]. The presence of oocysts may simply reflect passage through the gastrointestinal tract with no evidence of tissue infection [19]. *Cyclospora* spp. oocysts have also been detected in feces of dairy cattle based on molecular data [20]. Earlier molecular detection confirmed the microscopic finding of oocysts in dogs, chickens, and rhesus monkeys (*Macaca mulatta*) [21]. *Cyclospora-*like oocysts were observed in feces of several other species including carnivores, artiodactyla, and nonhuman primates from a zoological garden from Spain [16] by coprological analysis, however, no molecular methods or sequencing were performed and therefore, those oocysts could be from species other than *C. cayetanensis*. Other authors suggest that free living nematodes, insects, and rotifers could play a role in the spread of *Cyclospora* [3]. Together, these data seem to confirm that humans are the only natural host for *C. cayetanensis* and, thus, the major reservoir.

There are many aspects on *C. cayetanensis* epidemiology, life cycle, transmission and infection. Among others, many details of the development of *C. cayetanensis* stages are lacking—there is not enough information on the infective dose, when sporulation takes place, persistence in the environment, the role of water and soil on transmission and/or about the existence of potential reservoirs. Further prevalence studies are needed, as well as studies on population structure, and there is an urgent need for an effective genotyping method for source tracking in outbreak investigations. There are no animal models, and/or in vivo and in vitro culture systems to facilitate *C. cayetanensis* research. This review presents updated aspects on the biology, epidemiology, outbreaks, clinical symptoms, diagnosis, treatment, control and prevention of *C. cayetanensis* and outlines future research needs to fill the knowledge gaps for this parasite.

## 2. Morphology and Life Cycle

Unsporulated oocysts are spheroidal, 8–10 µm in diameter, with little or no size variation. Sporulation occurs outside the host. The oocyst wall is colorless, thin (<1 µm), and bilayered. Polar body and oocyst residuum are present [2]. Sporocysts are ovoidal, ~4 × 6 µm, and contain both Stieda and substieda bodies, and a large residuum. A sporulated oocyst contains two sporocysts, each with two sporozoites. The sporozoites are elongated, ~1 × 9 µm [1]. Sporozoites apparently lack crystalloid body or refractile bodies.

The life cycle of *C. cayetanensis* has not been fully described. The oocyst is the only stage definitively identified. Information regarding the endogenous developmental stages is based on biopsy specimens of patients [22,23,24,25,26]. Schizonts and gamonts were identified at the luminal end of jejunal enterocytes [24]. Presence of schizonts in intraepithelial enterocytes was reported in human immunodeficiency virus (HIV)-positive patients [26], and intracellular merozoites and binucleate schizonts were observed in enterocytes of terminal ileum and duodenum in a young HIV-positive woman [27].

**Figure 1:**
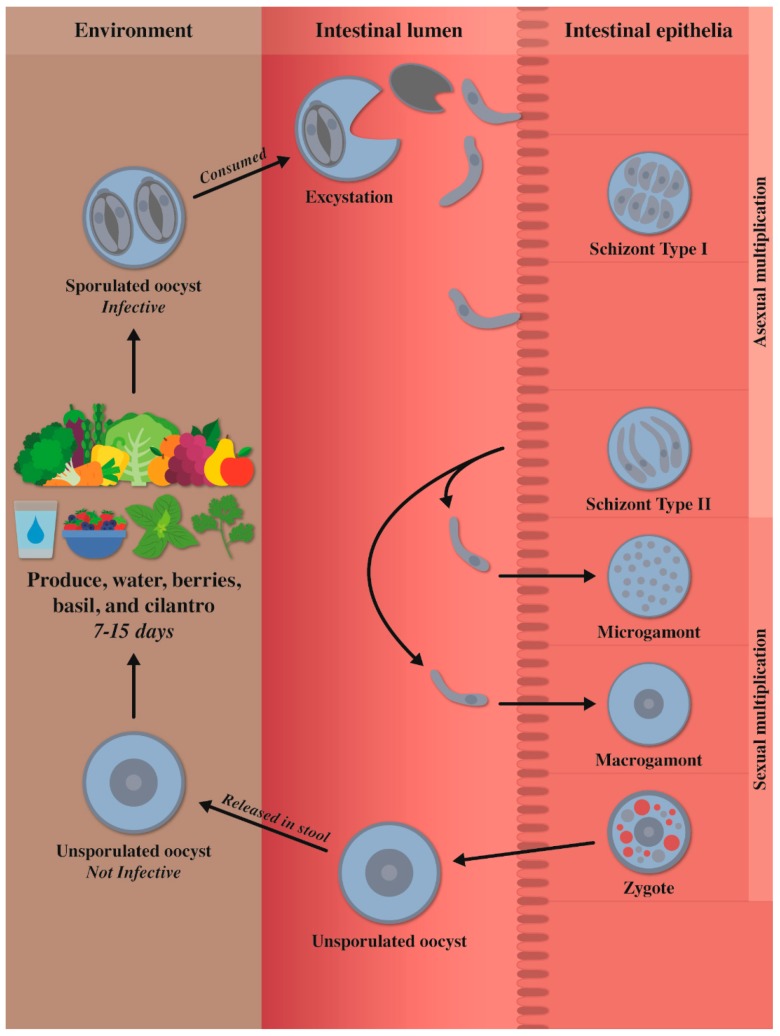
Life cycle of *Cyclospora cayetanensis.* **Caption:** If a susceptible human ingests sporulated oocysts in contaminated food or water, the sporozoites inside the sporocysts excyst in the gut lumen and invade enterocytes of the epithelium of duodenum and jejunum where the sporozoites transform into trophozoites. Trophozoites subsequently form 2 types of schizonts (asexual multiplication). Type I schizonts contain 8–12 merozoites. Type II schizonts contain 4 merozoites. Then, type II merozoites form gamonts (sexual multiplication). There are two types of gamonts: microgamonts and macrogamonts. Microgamonts fertilize macrogamonts to form the zygote. Oocysts then are formed in enterocytes and are excreted unsporulated in the feces. The prepatent period is thought to be around one week. Unsporulated oocysts are not infectious—they need to sporulate to became infective for a host. Under laboratory conditions, at 22 °C and 30 °C, sporulation will take between 7 and 14 days to occur outside the host. A sporulated oocyst contains two sporocysts, each with two sporozoites.

After the ingestion of sporulated oocysts in contaminated food, water, or soil by a host, sporozoites excyst in the gut lumen and invade the enterocytes of the epithelium of the duodenum and jejunum (Figure 1) [22,24], where the sporozoites transform into trophozoites which subsequently form two types of schizonts. Type I schizonts (size unknown) contain 8–12 small (3–4 µm long) merozoites. Type II schizonts contain 4 long (12–15 µm long) merozoites [24]. The type II merozoites form gamonts. The dimensions of gamonts are unknown. In the sexual multiplication, the microgamont fertilize the macrogamont to form the zygote; the flagella have not yet been identified. Oocysts are formed in enterocytes and excreted unsporulated in feces [24,25]. The prepatent period is thought to be one week [24].

Unsporulated oocysts have been reported in human sputum [28,29]. Cholecystitis and biliary involvement have been confirmed histologically in HIV-infected patients [30,31,32].

### Oocyst Sporulation

Under laboratory conditions, at 22 °C and 30 °C, *Cyclospora* oocysts stored in deionized water or potassium dichromate can sporulate in 7–14 days [33]. Sporulation was observed when oocysts were exposed to 37 °C for 4 days and 50 °C for 1 h. Storage at 4 °C or 37 °C for 14 days retarded sporulation [33] and only 12% of human-and baboon-derived *Cyclospora* spp. sporulated. Oocysts, previously stored at 4 °C for one to two months, sporulated when later stored for 6 to 7 days at 30 °C. The effects of temperature, including freezing and heating conditions, on the sporulation of *C. cayetanensis* were studied in dairy products and basil [34]. Sporulation occurred in those matrices at 23 °C, but it was inactivated when oocysts were exposed to extreme temperatures. No sporulation was observed at −70 °C, 70 °C, and 100 °C for either water or basil test samples, and there was no sporulation when dairy products were cooked at 70 °C, frozen at −70 °C for 1 h, or after exposure to −15 °C for 24 h [34]. Similarly, oocysts did not sporulate in basil kept at −20 °C for 2 days and in water for 4 days [34].

Pesticides, such as fungicides and insecticides and the combination of some of these products (captan 50% Wettable Powder (W.P.), benomyl 50% W.P., and diazinon 4E 47.5%) used at recommended concentration levels did not affect the sporulation of *C. cayetanensis* [35]. Oocysts from some patients with severe diarrhea may not sporulate (JP Dubey, own observation).

## 3. Transmission and Epidemiology

The modes of transmission of *C. cayetanensis* are still not completely documented, although fecal–oral transmission is the major route [36]. Direct person-to-person transmission is unlikely. Indirect transmission can occur if an infected person contaminates the environment, the oocysts sporulate under the right conditions, and then contaminated food and water are ingested. The role of soil in transmission has also been proposed [37]. The relative importance of these various modes of transmission and sources of infection is not known.

*Cyclospora cayetanensis* infection has been reported worldwide, in both developed and developing countries, but it is most common in tropical and subtropical areas. Initially identified endemic areas were Haiti, Guatemala, Peru and Nepal, where the first outbreaks were reported. Currently, endemic areas are considered to be Central and South America, several countries in the Middle East (Egypt, Turkey), the Indian subcontinent with Nepal, and South East Asia, including Indonesia [18,38,39].

In the 1990s, studies in susceptible populations in endemic areas showed prevalence levels in those particular groups around or higher than 10%. For example, 11.2% of 964 foreigners in Nepal [40], 11% of 450 HIV-infected patients in Haiti [41], 12.4% of 459 foreign residents in [42], and 18% of 144 in children in Peru [1] were infected. During the same period, prevalence ranged from 0.3 to 0.5% in the USA [43,44] and in the UK, where a total of 6151 stools were tested from 5374 patients and only 7 stools samples from 4 patients (0.1%) were positive [45].

In 2010, a review of previous studies on endemic areas from 22 countries (Mexico, Guatemala, Honduras, Brazil, Peru, Venezuela, Cuba, Turkey, Jordan, Saudi Arabia, China, Nepal, Bangladesh, Lao PDR, Thailand, Indonesia, Egypt, Nigeria, Uganda, Kenya, Tanzania and Mozambique) was published [46]. This review reported that the infection rates in those countries up to 2010 ranged from 0% to 13% (average 1.7%) in 47,642 apparently immunocompetent individuals, most with diarrhea, from endemic areas attending health care centers. Based on the same review, rates from matched asymptomatic controls varied from 0% to 4.2% (average 0.4%). In the same metadata analysis, higher prevalence rates were observed in immunocompromised persons; among 3340 immunocompromised persons, mostly HIV/AIDS patients with diarrhea, prevalence ranged from 0% to 36% (average 4.5%) [46]. The highest prevalence was observed in Peru (41.6%) [47]. Another systematic review of 27 studies from 14 sub-Saharan countries, revealed an overall prevalence of 18% [48]. A recent review gives detailed information on prevalence and geographical distribution of *C. cayetanensis* worldwide [49].

In studies published after 2010, the prevalence levels in endemic areas varied, ranging from 0.6% in Mexico [50] to 24.2% in Venezuela [51], with highly variable levels also in immunocompetent persons [from 0.5% of 2540 patients in Thailand [52] to 22.2% of 256 patients in India [53]]. Two recent studies in school children in Nepal reported lower prevalence levels (1.6% of 1392 and 3.9% of 507 children tested, respectively) [54,55]. In the most recent study in Mexico, prevalence levels were 2.9% of 277 samples from 104 participants of different ages [56].

### 3.1. Seasonality

*Cyclospora cayetanensis* infection is remarkably seasonal worldwide [57,58,59,60,61,62]. This seasonality varies by regions, most likely due to human activities, environmental contamination, and the optimal sporulation conditions in each area. The reasons for the apparent absence of symptomatic human infection for prolonged periods, where the parasite is present in the environment, and which biological conditions are needed for the survival of the parasites during these prolonged periods is unknown [63].

Factors such as rainfall, temperature, humidity, and perhaps photoperiod could affect the seasonality, which clearly cannot be related to rainfall alone, as there is a marked seasonal variation in very dry environments [59,64,65]. The incidence of *C. cayetanensis* infection increases in warm periods of maximal rainfall in countries such as Guatemala, Honduras, Mexico, Jordan, Nepal or China [40,50,55,58,59,61,62,66]. However, infection is more prevalent in the absence of rain, during the drier and hotter months of the year in Peru and Turkey [64,65,67]. In Haiti, infections occur during the driest and coolest times of the year, or during the cooler wet season in Indonesia [42,57]. In India, clinical cases were more frequent in the summer before the rainfall period [68]. Therefore, it is difficult to explain a common factor for the differences observed in seasonality. It should be considered that temperatures at the cooler time of the year in Haiti are closer to the warmest months of the year in Guatemala City and Kathmandu [57].

In Vietnam, contamination of produce increased before the rainy season [69] and in Guatemala clinical cases increased in April through June, coinciding with the raspberry harvest [58]. Furthermore, the seasonal pattern observed in endemic areas such as Mexico [50] coincides with maximal clinical prevalence in the USA [60,70].

### 3.2. Risk Factors

In general, children, foreigners, and immunocompromised patients in endemic developing countries will be the most vulnerable to *C. cayetanensis* infection. The main risk factors for cyclosporiasis in industrialized countries include international travel to cyclosporiasis-endemic areas and domestic consumption of contaminated food—mainly fresh produce imported from these regions. In most developed countries, the disease has been primarily associated with foodborne outbreaks.

There are likely be unknown risk factors, particularly in endemic areas, where studies have been skewed towards those with clinical symptoms [39].

#### 3.2.1. Contact with Animals

Several studies showed that contact with animals (e.g., dogs, chickens, ducks) was a risk factor for infection in endemic countries and increased the risk for human infection with *Cyclospora* [15,46,55,58,59,66,71,72]. As indicated earlier, the presence of oocysts in animals may simply reflect passage through the gastrointestinal tract, since to date there is no evidence of tissue infection in animals. However, even in this way, animals could aid in the dissemination of oocysts in the environment. However, this was not the case in Haiti [73], where key risk factors for infection with *Cyclospora* included using water from an artesian well, having a shallow, porous well and living in a stick and adobe house rather than a cement house. In that study, eating uncooked foods and having animals did not show any statistically significant correlation with infection [73].

#### 3.2.2. Age

In the developed world, cyclosporiasis has been observed in the general population regardless of age [60,70,74]. In endemic areas, on the other hand, young children are most affected by the disease [46,50,54,55,58,61,65,75,76]. Prevalence in children varied with region as well as in the studies within each region [46]. Prevalence ranged from 0% in Bangladesh, Kenya, Mozambique, or Tanzania to 33% (123/386) positive children in a cohort study in a community of Peru [59]. More recent studies (since 2010) have reported prevalence levels from 0.1% to 5.3% in children from Turkey, Kenya, India, Egypt, Mexico, Morocco and Nepal [50,54,55,77,78,79,80,81]. High prevalence (5.3% of 150 children tested) was observed in children in an intensive care hospital unit in Turkey [81]. The highest prevalence was reported in school children (16.5% of 415 pupils) in Madagascar [82]. In studies of malnourished children, 5.6% (2/36 children examined) were found to be infected in Egypt [83], while 0.9% were found to be infected in Guatemala (1/11 examined) [84].

The highest risk of infection and diarrhea in children occur in the first five years of life [46,55,59,64,85,86]. Infections in children <18 months of age are uncommon [85], which may be partially due to protection via maternal antibodies. In older children (>11 years) and adults, clinical symptoms are not always present [87,88]. The causes for this age distribution pattern are not clear, but may be related to predominant modes of exposure, shared foods and water from which very young children are relatively protected [39,59]. After an initial episode of cyclosporiasis, the likelihood of diarrhea and the duration of symptoms decreases significantly with each subsequent infection [59]. High percentages of asymptomatic carriers have been noted in endemic areas [46,52,55], suggesting that persistent contact with oocysts during the first years of age induces protective immunity against disease that will last into adulthood in endemic areas [38,89]. However, some outbreaks have been reported among local populations, such as naval recruits in Peru, [89,90] which challenge this theory. An explanation to these results would be that the acquired immunity is not long lasting and either diminishes over time [90] or that the geographic distribution of *C. cayetanensis* is unequal, leaving some populations unprotected [89]. It seems clear that the persistent contact with oocyst confers some resistance against the disease.

#### 3.2.3. Gender

Gender does not have a significant effect on the *Cyclospora* infection rate in different geographical areas [77,88,91].

#### 3.2.4. Socioeconomic Status

The epidemiology of *C. cayetanensis* infections is affected by socioeconomic status. Poverty and low socio-economic status are considered as risk factors for infection [36,88,92].

Cyclosporiasis is common in impoverished endemic areas where water and food sanitation are poor or non-existent, particularly affecting children [1,59]. A correlation between socioeconomic status and infection was observed in Venezuela [88]. In this country, most cases of cyclosporiasis were clustered in areas of extreme poverty where residences were without a latrine or toilet; contact with fecal-contaminated soil was also strongly associated with infection [88]. Higher rates of infection associated with deficient sanitary facilities were also observed in other endemic regions [54,73,91,93]. Even in developed countries like Germany, workers without proper toilet facilities were believed to be the source of an outbreak [94]. Studies in low socioeconomic areas have shown high prevalence, such as in Chennai, India (22.2% in 256 samples collected) [53], and in an indigenous Karina community in Venezuela (10% of 141 fecal samples collected) [95].

Socioeconomic status also influences the age pattern of infection by *C. cayetanensis*. In very poor areas of Peru, *C. cayetanensis* prevalence was highest in young children (aged 2–4 years); the infection was almost never detected after 11 years of age and adults usually did not have any clinical infection. In contrast, in middle to upper class families who lived in dwellings with suitable sanitation, children rarely appeared to become infected and infections occurred mainly in adults [36].

#### 3.2.5. Immunocompromised Hosts

*Cyclospora cayetanensis* affects both immunocompetent and immunocompromised persons. However, it is more severe in immunocompromised hosts, in particular in HIV-infected patients for whom it is an important cause of diarrhea [41,46,96,97]. Clinical illness due to *C. cayetanensis* in immunocompromised patients is prolonged, severe and is associated with a high rate of recurrence that can be attenuated with long-term suppressive therapy [30,41,98]. The average duration of diarrhea for HIV-infected patients is longer than that for HIV-negative patients (199 days vs. 57.2 days) [30] and other manifestations, such as acalculous cholecystitis has been reported in HIV-positive patients [30,32].

Since the 1990s, there have been many reports on *C. cayetanensis* infection in immunocompromised patients worldwide—mainly in AIDS/HIV-infected people, but also in immunosuppressed persons, including those undergoing cancer treatments [46,99,100]. In HIV-positive patients with chronic diarrhea, very different prevalence levels are reported in different parts of the world, with marked geographical variations, ranging from 0% in Cameroon or Zimbabwe [97,101] to 34.0% in Haiti [102]. However, most studies of *C. cayetanensis* infection in HIV-positive patients up to 2010 showed levels of prevalence around or lower than 4% (reviewed by [46]), and similarly, levels around 4% have also been observed in studies since 2010 [103,104,105,106]. Detection of the parasite was mostly based on microscopy, but in a few of the studies in HIV-positive individuals, the presence of *C. cayetanensis* DNA was detected by molecular techniques [102,107,108]. Some studies showed correlation between mean CD4+ T cell counts and *C. cayetanensis* infection [26,109], although some other studies have shown no association with CD4+ T cell counts among HIV positive patients with *C. cayetanensis* infection [106,108]. Similarly, in children, a higher prevalence was observed in immunosuppressed children (7.8%, 13 of 166 children) compared to the immunocompetent children tested in the same study (2.1%, 3 of 142) [78].

A high prevalence is generally observed in patients immunosuppressed with pathologies such as Hodgkin’s lymphoma and acute lymphoblastic leukemia (24.5% of 49 examined) [107]. In a study in cancer patients receiving chemotherapy in Saudi Arabia, a very high prevalence was observed (52% of 54 patients) and a particularly high *C. cayetanensis* prevalence was observed in patients with lymphomas [110].

#### 3.2.6. Resident Foreigners, Expatriates and Traveler’s Diarrhea

Resident foreigners and travelers to endemic areas are at a high risk of infection with clinical disease and are more susceptible to the disease than the indigenous population, including children [42].

Between 1997 and 2008, 33.5% of laboratory confirmed cases of infection in the USA were related to travel [70]. In Canada, 71% of reported cyclosporiasis cases between 2000–2010 were in travelers [111]. The remainder were domestically acquired, presumably foodborne.

Most cases of cyclosporiasis reported in Europe and Australia have been acquired by visitors in regions where the parasite is endemic [3]. Countries visited were: Bali, Bangladesh, Bolivia, Bulgaria, Cambodia, China, Colombia, Costa Rica, Cuba, the Dominican Republic, Gabon, the Greek Islands, Guatemala, Hong-Kong, India, Indonesia, Java, Lebanon, Madagascar, Malaysia, Mexico, Morocco, Nepal, Nigeria, Pakistan, Peru, Puerto Rico, Singapore, the Solomon Islands, South-East Asia, Sri Lanka, Thailand, Turkey and Vietnam [3]. In some European countries, including Spain [112,113,114], the UK [45,115,116,117]; Poland [118] or Germany [119], all *C. cayetanensis* cases diagnosed were related to traveler’s diarrhea. In the Czech Republic, out of six cases reported, five had traveled to endemic areas while the other patient did not have any travel history outside the country [120]. Cases of travelers from the USA were reported after returning from Mexico, Thailand, Haiti and Puerto Rico [3].

There have been several outbreaks in expatriates living in developing countries such as Nepal and Indonesia [3] and in foreign visitors to endemic countries [113,115,118,121].

#### 3.2.7. Consumption of Contaminated Food

A foodborne infection of *C. cayetanensis* is the main route of transmission of the parasite [122,123,124]. Contaminated fresh produce—such as berries and leafy greens, which are difficult to clean thoroughly and are consumed without prior processing to inactivate or remove the oocysts—are the major source of infection. Pasteurized foods or foods thoroughly heated before consumption have not been associated with illness [125].

*Cyclospora cayetanensis* contamination has been reported in fresh produce surveillance studies in several countries, mainly in endemic areas (Table 1). To date, no surveillance studies have been published in the USA. The cases identified in the USA prior to 1995 were all thought to be linked to imported food or were associated with people who had traveled to areas where the disease was endemic. During the period of 2004–2009, 37.8% (70/185) of the cases were classified as domestically acquired [60]. In 2018, for the first time, *C. cayetanensis* was identified in produce grown in the USA (Press announcement, Statement from Food and Drug Administration (FDA) commissioner on 18 September 2018).

*Cyclospora* spp. in references [69,126,127,128,131,132,133,139] and *Cyclospora cayetanensis* in references [71,76,129,130,134,135,136,137,138].

Recent studies have shown *C. cayetanensis* contamination in ready to eat and pre-packaged/bulked vegetable products in Canada and Europe [128,129,135,136]. The contamination of ready to eat and pre-packaged/bulked vegetables is an indication that the current sanitation processes do not guarantee food safety when dealing with certain parasites of fecal origin [136]. The global trade of foods may play a significant role in the transmission of *C. cayetanensis* in developed countries, such as the USA, considering that some of the outbreak cases have been traced back to fresh produce imported from developing regions [140].

Raspberries, blackberries, mesclun (mixture of varieties of lettuce), bagged mixed greens, snow and snap peas, cilantro, and basil are produce commodities implicated in numerous food-borne *C. cayetanensis* outbreaks worldwide (Table 2) [94,99,141,142,143,144,145,146,147,148].

Historically, cilantro and raspberries have been two of the main matrices linked to cyclosporiasis outbreaks in North America [99,141,173,179,180]. Contaminated Guatemalan raspberries were responsible for high-profile foodborne outbreaks in the USA and Canada in the 1990s [99,141,151,153,166,169]. Recently, the main cause of outbreaks in both the USA and Canada has been consumption of contaminated cilantro [154,173,175]. The outbreaks occurring in the USA from 2013–2015 were linked to imported cilantro from Puebla, Mexico [173,175]; cilantro continues to be a source of *C. cayetanensis* infection in the USA, since multiple cilantro-associated clusters, with 53 laboratory-confirmed cases—mostly related to Mexican-style restaurants—were identified in 2018 [175].

Basil is another leafy green that has been linked to outbreaks in the USA and Canada [142,154,155,156,170]. The most recent outbreaks associated with basil were two clusters of eight confirmed cases identified in two states in the USA in 2018 and one multistate outbreak in 2019, with 205 lab-confirmed cases from 11 states linked to fresh basil imported from Mexico (as of August 28th, 2019). Contaminated basil was found in surveillance studies in Vietnam and Nepal [69,76].

Salad greens, such as lettuce, were linked to outbreaks in the USA and Europe [94,99,146,147,181] and romaine-carrot mixes were linked to a recent outbreak in 2018 due to the consumption of salads from a fast-food restaurant chain in the USA [177]. Lettuce contaminated with *Cyclospora* spp. and/or *C. cayetanensis* oocysts has been found in many countries (Canada, Costa Rica, Egypt, Ethiopia, Ghana, Italy, Nepal, Peru, Venezuela or Vietnam) (Table 1). Contaminated snow peas were linked to an outbreak in the USA [172] and Guatemalan imported snap peas were linked to two separate outbreaks in Europe and Canada [143,148].

In 2016, a small outbreak in the USA was linked to coleslaw (cabbage and carrot mix with dressing) [140,174]. In 2018, in the most recent outbreak in the USA, pre-packaged vegetables trays containing carrots, broccoli, cauliflower, and dill dip were the cause of [178].

For many outbreaks, the contaminated food source was not identified. The identification of food items that serve as vehicles in cyclosporiasis outbreaks represents a major challenge. The unknown incubation period for *C. cayetanensis* infection, the short shelf-life of implicated commodities (i.e., fresh produce), and the complex epidemiological investigations required to identify the contaminated produce item present in a dish with multiple ingredients, are among the factors that hamper these investigations [140]. Fresh produce is often consumed raw and with little or no washing and may become contaminated by food handlers or through crop irrigation with untreated water. Factors including poor worker hygiene practices, contaminated soil, and contaminated agricultural water could play a role in contamination. In many instances, the fresh produce linked to outbreaks may be a component of complex dishes with many produce ingredients, which makes detection more difficult [63,140].

#### 3.2.8. Waterborne Infection of *C. cayetanensis*

Water contaminated with fecal matter may act as a vehicle of transmission for *C. cayetanensis* infection. The source of drinking water has been determined as a risk factor for cyclosporiasis in endemic areas [54,55,66,72,73]. More importantly, *C. cayetanensis* oocysts have been detected in several types of water (Table 2)—including chlorinated water, and wastewater in endemic areas and in non-endemic areas—which suggests the potential spread of the parasite via drinking and recreational water. *Cyclospora cayetanensis* oocysts have been detected in different sources of water in many endemic countries such as Guatemala, Haiti, Ghana, Vietnam, and Egypt, among others (Table 3). In early studies in Nepal, chlorinated drinking water and sewage were found to be contaminated [71,161]. In Nepal, a higher risk of enteric parasites was observed in school children who drank direct tap water instead of boiled water [54]. In Peru, the consumption of unchlorinated drinking water was associated with cyclosporiasis [13] and wastewater was found to be positive, both microscopically and by molecular methods in a Peruvian impoverished area of improvised housing [182].

*Cyclospora cayetanensis* contaminated water has also been found in developed countries. In Italy, treated wastewater, tap water, and well water was found, by molecular methods, to be contaminated by *C. cayetanensis* [188]. In Spain, drinking water treatment plants (DWTP), wastewater treatment plants (WWTP) and rivers were tested—oocysts were present in one of the rivers investigated [190]. In the USA, WWTP influent (25% positive samples, 6/24) and WWTP effluent (12.5% positive samples, 3/24) were found to be contaminated in Arizona [193].

The potential for the oocysts to be transmitted by wastewater contamination of drinking or irrigation water has also been indicated [72,182,190,193,195].

Some *C. cayetanensis* outbreaks are waterborne, but the source of contamination has not been established and other sources have not been ruled out [40,89,90,99,118,160,161,163,165,196]. In an outbreak in Turkey, co-infection with *C. cayetanensis* during a waterborne outbreak of *Cryptosporidium* occurred [163].

In countries where *C. cayetanensis* is endemic and water and sewage treatment systems are insufficient, waterborne oocysts are a likely source of infection. Oocysts can pass through physical barriers and are not affected by chlorine and other water disinfectants [197]. Cyclosporiasis was associated with consumption of unchlorinated water and drinking water that was inadequately filtered [13,161,198]. Oocysts also seem able to survive treatment protocols used in WWTP, since both influent and effluent treated water showed similar frequencies of *Cyclospora* spp. in these plants [190].

*Cyclospora cayetanensis* can contaminate plant crops via different pathways, including black water used for the irrigation or spraying of crops, contact with contaminated soil, infected food handlers, or hands that have been in contact with contaminated soil [125]. Irrigation of crops by using untreated or poorly treated water is a likely source of contamination for fruits and vegetables [199]. Contaminated water used for applying fertilizers and for washing and processing foods are also likely sources of foodborne transmission. In addition, recreational exposure to water contaminated with *C. cayetanensis* oocysts may also be a source of infection [189].

A few studies evaluated the role of shellfish in transmission of *Cyclospora.* The Asian freshwater clam (*Corbicula fluminea*) could be used as a biological indicator for recovery of *C. cayetanensis* oocysts from water [200]. *Cyclospora cayetanensis* oocysts have been found in bivalves, gandofli (*Caelatura Iaronia pruneri*) in Alexandria, Egypt [201] and *C. cayetanensis* DNA was detected by real time PCR with High Resolution Melting (HRM) analysis in mussels (*Mytilus galloprovincialis*) (26.4% of 53) collected at the Aegean coast of Turkey [202]. Using mollusk bivalves as biological sentinels, *C. cayetanensis* oocysts were also recently observed in Tunisian coastal waters [203]. The presence of *C. cayetanensis* in shellfish could indicate that freshwater run-off from land could carry oocysts into the marine ecosystem [39].

#### 3.2.9. Soil as a Source of Infection and/or Transmission for *C. cayetanensis* Oocysts

Soil is a potential and possibly important mode of transmission and source of infection for *C. cayetanensis* [37,88]. The contamination of soils by inadequate defecation disposal might be a significant determinant for infection. Some studies have included contact with soil as a risk factor for *C. cayetanensis* infections, in both developing and developed countries [88,197]. In Venezuela, most cases of *C. cayetanensis* were clustered in the areas of extreme poverty where living in a hut, not having a toilet, and having contact with soil contaminated with human feces were strongly associated with infection [88]. *Cyclospora cayetanensis* was more prevalent where agricultural work and lack of hand washing were present [54]. More recently, soil was found to be positive for oocysts in Italy (11.8% positive samples, 6/51) [188]. Higher rates of infection have been noted in additional areas where risk factors such as deficient sanitary facilities, poor personal hygiene, and soil contaminated with human feces were present [73,91,93].

## 4. Outbreaks Due to *C. cayetanensis*

Table 2 presents a summary of the outbreaks associated with *C. cayetanensis* worldwide, with possible routes of transmission. More than 11,500 cases have been reported to date in North America (Canada and USA). Of those, only in the USA in the last four years (2016–2019) have there been almost 4500 cases and counting, since the outbreak season is not finished for the current year. In other areas of the world (excluding North America), more than 2500 cases have been reported. Considering that many countries do not include the parasite in their diagnostic protocols, these numbers are clearly an underestimate of the real cases related to C*. cayetanensis* infection.

Most outbreaks due to *C. cayetanensis* described to date have been related to fresh produce consumption. *Cyclospora cayetanensis* outbreaks have been mostly reported in North America, probably due to better detection methods and disease surveillance that have helped in tracking outbreaks.

In the mid-1990s, *C. cayetanensis* was recognized as the causative agent of multistate outbreaks of diarrheal illness in the USA and Canada. The outbreak that brought cyclosporiasis to importance in North America, affecting both USA and Canada, occurred in the spring of 1996 and was due to the consumption of fresh raspberries imported from Guatemala. A total of 1465 cases were reported by 20 states and the District of Columbia in the USA, and in two Canadian provinces [151]. Until 2000, 90% of cases in the USA have been food-related, with approximately 15,000 foodborne cases estimated annually [122]. Infections were linked to fresh produce, mostly berries and leafy vegetables imported from Mexico and Central America into the USA and Canada (Table 2). Between 1990 and 2000, nearly all reported outbreaks in the USA and Canada were associated with food and mostly related to Guatemalan raspberries [38,99,141,151,153,166,169]. In the 1990s, most of the outbreaks of cyclosporiasis were reported from North America (USA and Canada) and three were in Nepal [40,99,154,160,161]. Since 2000, at least 31 outbreaks have been reported worldwide, 18 of those in North America [39]. The 2013 multistate outbreaks in the USA with 631 laboratory-confirmed cases contributed to the largest annual number of cases of cyclosporiasis in the USA since 1997 until that year [173]. The 2014 and 2015 multistate outbreaks in this country involved 304 and 546 confirmed cases in 19 and 31 states, respectively [171]. In 2016, a restaurant-associated sub-cluster of cyclosporiasis in Texas was epidemiologically linked to consumption of coleslaw containing shredded carrots and cabbage [140,174]. In 2017, although many cases (1,065) were diagnosed in the USA, only a few of them were clustered as outbreaks and linked to a specific commodity (e.g., 38 cases in Texas linked to the consumption of scallions (i.e., green onions) and 6 cases in Florida suspected of being caused by the consumption of berries) [171] (Table 2). In 2018, the incidence of *C. cayetanensis* infections increased markedly, in part related to large outbreaks associated with produce [204]. On the other hand, two main outbreaks and several smaller ones took place in 2018 in the USA. An outbreak in four states, with 250 laboratory-confirmed cases of *Cyclospora* infection, was linked to the consumption of pre-packaged vegetable trays containing broccoli, cauliflower, carrots, and dill dip from supermarkets. Eight people were hospitalized. No deaths were reported. It was not possible to determine if an individual component of the vegetable trays was the likely vehicle of infection [178]. A separate outbreak involved 511 laboratory-confirmed cases of *Cyclospora* infections in people from 16 states who reported consuming a variety of salads from McDonald’s restaurants in the Midwest. Twenty-four people were hospitalized; no deaths were reported. Epidemiologic and traceback evidence indicated that salads purchased from McDonald’s restaurants were one likely source of this outbreak [176,177]. The Food and Drug Administration (FDA) confirmed the presence of *Cyclospora* in an unused package of romaine lettuce and carrot mix (distributed to McDonald’s by the Fresh Express processor in Streamwood, Illinois, USA) [177].

In 2018, from the total of 2299 confirmed cases, as of October 2018, approximately 35% ill people were associated with either of the two outbreaks indicated: vegetable trays and salads [175,176]. In addition, two basil-associated clusters of eight confirmed cases in two states and multiple cilantro-associated clusters in Mexican-style restaurants including 53 confirmed cases associated to three clusters in the Midwest, took place. Many cases of cyclosporiasis could not be directly linked to any outbreak [175,176]. In 2019, as of August 28th, 1,696 laboratory-confirmed cases of cyclosporiasis were reported to the Centers for Disease Control and Prevention (CDC) by 33 states, District of Columbia and New York City in people who became ill since May 1, 2019 and who had no history of international travel during the 14-day period before illness onset. At least 92 people were hospitalized.

Waterborne outbreaks have been suspected (see Section 3.2.8). In one outbreak reported in the USA [165], the fixing of a water pump and refilling storage tanks in a penthouse area of the dormitory of a physician was thought to be the cause and this would have been the first described waterborne outbreak in the USA. However, examination of water samples did not reveal *Cyclospora* oocysts and the mean time of incubation did not seem right [99]. Some outbreaks related to cruises have been documented. The most important outbreak was related to two consecutive voyage cruises departing from Australia in 2010 in which 266 people were affected (73 were laboratory-confirmed) and lettuce was suspected as the cause of the outbreak [144].

Several outbreaks have been reported in expatriates or visitors to developing countries (Section 3.2.6). The most important and recent outbreaks were reported in (1) Visitors from the Riviera Maya region of Mexico in 2015, coming back to United Kingdom (UK) (79 cases) and Canada (97 cases) [115]. (2) After a scientific meeting of a group of Dutch people in Bogor, Indonesia in which around 50% of the investigated participants (14 of a total of 29) were positive for *C. cayetanensis* [121], and (3) in Spaniard workers who traveled for international aid-related purposes to Guatemala [113]. A small outbreak was reported in three Polish businessmen who traveled to Indonesia [118].

Interestingly, cyclosporiasis outbreaks have also been reported in local populations of developing areas. The most recent outbreaks in local populations took place in Mexico [162], Colombia [159], Peru [89,90] and Turkey [164] (Table 2). The outbreak in Turkey seemed to be due to insufficiently washed produce in an extremely dry summer [164]. The cases in Mexico and Colombia affected people attending weddings and religious ceremonies [159,162]. Two separate outbreaks of cyclosporiasis occurred in local endemic populations of naval recruits in Peru [89,90] and raised questions about immunity to *C. cayetanensis* in endemic areas [90].

## 5. Clinical Symptoms and Pathogenesis

The main symptoms of *C. cayetanensis* infection are voluminous watery diarrhea, abdominal cramps, nausea, low grade fever, fatigue and weight loss [3]. Although the disease is self-limiting in most of the immunocompetent patients, it may present as a severe, protracted or chronic diarrhea in immunocompromised patients [3].

The clinical presentation in endemic settings shows differences by age, with elderly persons and young children having more severe clinical symptoms, while infections are milder in older children and adults [25,205,206]. In addition, asymptomatic infections are frequent in endemic areas [95,207]. The severity and duration of infection tend to become milder after repeated episodes [3]. The median incubation period is around seven days [3,151,167]. The average duration of diarrhea is longer in HIV-positive patients than in HIV-negative patients (199 days and 57.2 days, respectively) [3,208,209]. Some syndromes have been reported following *Cyclospora* infection: Guillain–Barre syndrome [210] and reactive arthritis syndrome (formerly Reiter syndrome) [211]. Another clinical manifestation of *C. cayetanensis* infection in HIV-positive patients is biliary disease, acalculous cholecystitis and cholangitis in AIDS patients [30,31,32]. The pathogenesis of biliary infections is unknown. Presumably, some sporozoites from intestinal lumen travel to bile ducts and initiate the development of *Cyclospora* there; extra-biliary infections have not been reported.

The pathogenesis underlying these symptoms has not been defined. Jejunal biopsies have shown mucosal alterations in intestinal villi, diffuse edema and infiltration by inflammatory cells, reactive hyperemia, vascular dilation and congestion of capillaries in the presence of the parasite, which are compatible with inflammation of the upper intestinal tract [24].

## 6. Diagnosis

### 6.1. Serological Studies

Currently, no serological assays are commercially available. In a study, specific IgG and IgM antibodies in individuals with oocysts were tested by ELISA [93].

### 6.2. Oocyst Detection in Clinical Samples

#### 6.2.1. Oocyst Testing

**Figure 2:**
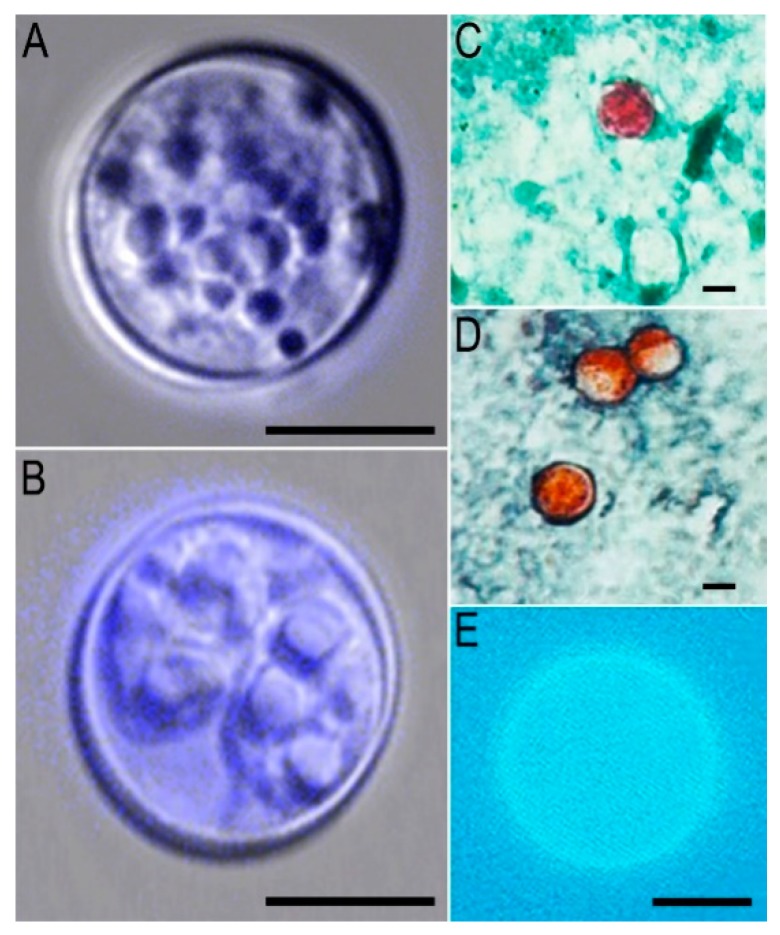
Oocysts of *Cyclospora cayetanensis.* Caption: *Cyclospora cayetanensis* oocysts. (**A**,**B**), unstained, differential Interference contrast, (**C**) acid-fast stain, (**D**) hot-safranin stain, (**E**), ultraviolet fluorescence microscopy. Oocyst in B is sporulated. Bars in A-E = 5 µm. Images (**C**–**E**) were from public images from DPDx CDC.

Multiple stool samples should be examined to rule out a *C. cayetanensis* infection. Three stool specimens collected on alternate days within a 10-day period should be examined to achieve >95% detection rate [212]. Wet smears, with or without iodine, may be used to detect *C. cayetanensis* oocysts. Concentration techniques can be used such as formalin-ether sedimentation or flotation using sucrose solution for detection of small number of *Cyclospora* oocysts in stool samples. Due to the small size (8–10 µm) and round shape of the oocysts, they can be confused with amoebae or inflammatory cells, and alternative microscopic methods are required to achieve a higher detection sensitivity [212] (Figure 2A,B).

#### 6.2.2. Modified Acid-Fast Staining

*Cyclospora*, *Cryptosporidium*, and *Cystoisospora* oocyst walls contain acid-fast lipids [212], which is a common property and makes acid-fast staining relevant for the screening of these three parasites in a single test. Although modified acid-fast staining can be used to identify these organisms, in *Cyclospora*, variable levels of dye uptake may result in ghost cells, or poorly stained cells, along with well stained ones [3,212,213] (Figure 2C). There have been minor variations in modified acid-fast stains to improve *Cyclospora* detection. One of the favored modifications is to use 1% H_2_SO_4_ as a decolorizer without alcohol [212]. Another variation is the addition of dimethyl sulfoxide (DMSO) to the phenol-basic fuchsine and the incorporation of acetic acid with malachite green as a combined decolorizer-counter stain to achieve better penetration for the visualization of the internal structures of the oocysts [212].

#### 6.2.3. Heated Safranin Staining

This staining was initially used for *Cryptosporidium* identification [214]. A modified heated safranin stain was reported to uniformly stain *Cyclospora* oocysts a brilliant reddish-orange color [214,215,216] (Figure 2D). Safranin staining showed agreement in sensitivity and specificity with Zielh Nielsen acid-fast staining technique [217].

#### 6.2.4. Auto Fluorescence

The oocysts of *Cyclospora, Cystoisospora* and *Cryptosporidium* have auto fluorescence properties [212,214,218]. Strong auto fluorescence of *Cyclospora* oocysts is a useful microscopic test for identification [212,214,219] (Figure 2E). *Cyclospora* appears blue when exposed to 365 nm UV light [220] and looks green under 450–490 nm excitation [221]. *Cryptosporidium* appears violet when exposed to 365 nm UV light, and green under 405–436 nm excitation [220].

### 6.3. Molecular Detection and Characterization

Molecular methods have various strengths for the diagnosis of cyclosporiasis, including the ability to simultaneously detect several pathogens using multiplex platforms, rapid assessment, and high sensitivity.

In a more recent study in Shangai, China, *C. cayetanensis* was not detected in any of the stool specimens by traditional microscopy, whereas five stool specimens (1.72%, 5/291) were positive by PCR [62]. Although several conventional, nested, [222,223,224], and real-time PCR assays (including multiplex PCR assays [225,226,227,228,229,230,231], among others) have been developed over the years, until recently, no commercially available molecular diagnostic test was available for use in clinical laboratory settings. The BioFire FilmArray gastrointestinal (GI) panel (BioMerieux) is the only commercially available molecular diagnosis product capable of detecting *C. cayetanensis*. This fully automated system can detect 12 enteric bacterial pathogens, five groups of viruses, and the protozoan parasites *C. cayetanensis*, *Cryptosporidium, Giardia*, and *Entamoeba histolytica*. After a simple sample loading process, fully automated cell lysis, nucleic acid purification, and high order multiplex PCR reactions occur in a pouch that contains all the reagents in microfluidics chambers where the FilmArray reactions and analysis occur. The multicenter evaluation of the BioFire FilmArray GI panel was performed on 1556 clinical stool samples. Performance of the FilmArray was compared with traditional and molecular assays to determine the sensitivities and specificities of the four protozoans in the GI panel. This study showed that both sensitivity and specificity for the *C. cayetanensis* assay were 100% in the FilmArray GI panel [232]. Recently, in a clinical practice setting, results using the BioFire FilmArray GI panel were found to be reproducible (34/35, 97%) [233].

The whole genome sequence of the parasite [234,235,236], mitochondrial, and apicoplast genome sequencing were recently achieved [237,238,239,240,241,242,243] and will be extremely useful in the development of new diagnostic assays for *C. cayetanensis*, including genotyping the parasite. A multilocus sequence typing (MLST) method, based on five microsatellite markers amplified by nested PCR, was described [244,245,246]. This method was evaluated in *C. cayetanensis*-positive stool specimens from 54 patients, including 51 from the United States [247]. Poor discriminatory power was found, and many specimens had what appeared to be a mixture of sequence types at one of the loci. The authors suggested that an alternative approach to MLST could be to perform Next Generation Sequencing (NGS) [247].

The detection of *C. cayetanensis* in produce is a challenge. Usually low numbers of oocysts are detected in naturally contaminated produce and the methods used for clinical samples are not always extrapolatable for detection in produce. One very important step is an efficient recovery of oocysts from fresh produce after careful washing. For the recovery of oocysts, different washing solutions have been used [248,249,250]. Of those, a commercial laboratory detergent (Alconox^®^) demonstrated an improved recovery compared to other solutions [248]. The use of lectin-coated paramagnetic beads for isolation of *Cyclospora* oocysts from fruits and vegetables was described, but no significant differences in recovery efficiencies could be detected with or without this procedure [251]. The isolation and concentration of *C. cayetanensis* oocysts using antibody-specific coated beads, as used for the detection of *Cryptosporidium parvum*, will likely improve efficiency, but antibodies are not yet commercially available. In recent years, several molecular techniques—including quantitative PCR (qPCR)—have been developed for detection in fresh produce [179,250,252,253]. As few as three oocysts per gram of fruit, or five oocysts per gram of herbs or green onions, were reliably detected by qPCR melting curve analyses [250]. Furthermore, as low as five oocysts in raspberries, cilantro, parsley, basil and carrots were detected by qPCR using an FDA validated technique [140,179,230].

A validated wash protocol for the recovery of *C. cayetanensis* oocysts from fresh produce, together with DNA extraction and a specific real time PCR for the detection of *C. cayetanensis* DNA, was recently published in the Bacteriological Analytical Manual (Chapter 19B) of the FDA [254]. The wash protocol for fresh produce is performed in a filter bag (BagPage^®^+) by adding 0.1% Alconox^®^ detergent as a wash solution. Bags containing leafy greens or sturdy vegetables (but not those containing fragile matrices such as berries) are massaged gently with fingertips a few times to remove most of the air. Bags containing berries should be sealed without massaging and without removing air. Once sealed, the bags containing leafy greens are placed flat in a tray on a rocker platform for 30 min at 85 rpm at room temperature, inverting the bags after 15 min; bags containing berries are placed upright in the tray and slowly rocked on a rocker platform for 30 min at a lower speed. Afterwards, the supernatant from the filtrate side of each filter bag is transferred to individual centrifuge tubes and sequential centrifugations are performed to recover, pool, and concentrate the wash debris. The wash debris pellets may then be stored at 4 °C for up to 24 h or frozen at −20 °C for longer periods prior to DNA isolation [254].

Purified oocysts from fecal samples are required for experimental seeding for the validation of detection techniques in produce, or for setting up other molecular sequencing and typing techniques directly from oocysts. Discontinuous percoll gradients [255] as well as a detachment solution, renocal-sucrose gradient sedimentation, and flow cytometry [256] have been used in the isolation of *C. cayetanensis* oocysts. A recent study used discontinuous density gradients in the presence of a detergent and flow cytometry to obtain highly purified oocysts for use in genomic studies [257].

Currently, the viability of the parasite can only be assessed by analysis of the sporulation rates of the oocysts. Techniques, such as vital dye assays (e.g., DAPI) are not commercially available. An electrorotation method, based on changes in the morphology and physicochemical properties of oocysts upon rotation, has been described [258], but the technique is complex and only used in research settings.

## 7. Treatment

Trimetoprim-sulfamethoxazole (TMP-SMX) is the antibiotic of choice used for the treatment of cyclosporiasis [41,64,75,98]) and is effective for both immunocompetent and immunocompromised patients [259]. Ciprofloxacin can be used as an alternative therapy in patients with sulfonamide allergies, although is not considered as effective as TMP-SMX [98]. When ciprofloxacin treatment was not effective, TMP-SMX was subsequently used and successful [27]. Other antibiotics such as azithromycin, nalidixic acid, diloxanide fluorate, tinidazole, norfloxacin, quinacrine [3,160]), have been tested for cyclosporisis treatment without success.

A new thazolide treatment, nitazoxanide, was used in a patient who did not respond to ciprofloxacin and was allergic to sulfonamides—seven days later, symptoms improved [260]. Nitazoxanide was used for treatment of mixed parasite infections with both intestinal protozoa and helminths, including *C. cayetanensis*. The efficacy for cyclosporiasis ranged from 71% to 87% in three treatment trials. Nitazoxanide was very well tolerated, with no serious adverse effects reported [261].

## 8. Control and Prevention

Infections of *C. cayetanensis* could be prevented by improved personal hygiene and sanitary conditions to eliminate possible fecal–oral transmission from contaminated food, water, and possible environmental samples in endemic areas. Infection could also be prevented by avoiding consumption of raw fresh produce, particularly in endemic areas.

There are some exploratory methods to remove or inactivate oocysts in fresh fruits and raw vegetables. Studies conducted on other coccidia may shed light on the control and prevention of *C. cayetanensis*. Using the chicken coccidian, *Eimeria acervulina*, gamma-irradiation, at 1.0 KGy and higher, was effective in the decontamination of raspberries [262]. Using the same surrogate, hydrostatic pressure (HPP) (550 MPa at 40 °C for 2 min) in raspberries and basil appeared effective, since broiler chickens that were fed the contaminated produce with treated oocysts were asymptomatic and did not excrete oocysts [263]. Sodium dichloroisocyanurate (NaDCC) has been used in disinfection studies against common intestinal protozoa, including *C. cayetanensis*, where raw vegetables and fruits were dipped into NaDCC solution (1g/liter) and parasite numbers were reduced [264]. Treatment of *C. cayetanensis* oocysts with magnesium oxide nanoparticles showed significant reductions in sporulation rates compared to untreated oocysts and could be used safely as a preventive agent in food and water disinfectant treatments [265]. Water used for drinking, food preparation, and washing of fresh produce to be eaten raw, should be boiled or filtered. As indicated earlier, *C. cayetanensis*, and coccidia in general, are highly resistant to sanitizers and disinfectants.

Controlling sources of contamination in the field, in packing houses, and from farm workers is the key to preventing *C. cayetanensis* infection, particularly in endemic areas. Access to toilet facilities, thorough hand washing, and the proper disposal and treatment of human sewage are essential. Workers having any symptoms of gastro-enteritis should not be allowed contact with vegetables or food. Other aspects of control and prevention used for other coccidian infections may also be applicable to *C. cayetanensis.* There is no vaccine to protect humans against this coccidian infection.

## 9. Conclusions and Future Research Needed

The importance of *Cyclospora cayetanensis* as a major cause of food-borne outbreaks is increasing in developed countries and remains an important cause of diarrhea in children in developing countries. Recently, newly developed, highly sensitive and specific molecular methods have facilitated clinical diagnosis, and the detection of oocysts in foods. Due to the absence of animal models and in vitro culture systems, only very limited biological material—namely oocysts isolated from clinical stool samples—is available for research, which presents a major bottleneck for research activities. The development of animal models, in vivo and in vitro culture systems is critical to facilitate *C. cayetanensis* research. Improved methods for the isolation of a small number of oocysts from diverse food matrices, and from water and soil samples are required. Next Generation Sequencing technologies have provided a way to sequence nuclear and organellar genomes of *C. cayetanensis* in the recent years. This major development has opened a door for method development for the molecular/genomic typing of the organism for use in outbreak investigations for source tracking. In addition, further research needs to be conducted to understand many epidemiological aspects of this infection, and methods will need to be developed to analyze the viability of this coccidian parasite in the environment and in food.

## Figures and Tables

**Table 1:** Positive reports and surveillance studies of *Cyclospora* spp. and/or *C. cayetanensis* oocysts detection in fresh produce worldwide.

Location	% (No. Positive/Total Samples Analyzed)	Food Type	References
Cambodia	8.3 (3/36)	Water spinach	[126]
Costa Rica	8.0 (2/25)	Lettuce	[127]
Canada	1.7 (9/544)	Precut salads and leafy greens	[128]
Canada	0.5 (6/1171)	Arugula/baby arugula, baby spinach and spring mix	[129]
Egypt	16 (4/25)	Lettuce heads	[130]
Egypt	21.3 (64/300)	Rocket, lettuce, parsley, leek, green onion	[131]
Egypt	25.7 (9/35)	Fresh strawberry juice	[132]
Ethiopia	6.9 (25/360)	Vegetables and raw fruits (avocado, lettuce, cabbage, carrot, tomato, banana and mango) *	[133]
Ghana	5.1 (20/395)	Cabbage, green pepper, onion, tomato, lettuce	[134]
Italy	12.2 (6/49)	Fennel, cucumber, celery, tomato	[135]
Italy	1.3 (8/648)	Ready to eat-packaged salads	[136]
Korea	1.2 (5/404)	Winter grown cabbage, sprouts, blueberries, and cherry tomatoes	[137]
Nepal	Number not indicated	Cabbage, lettuce, mustard leaves	[71]
Nepal	Number not indicated	Lettuce, spinach, mustard and basil leaves	[76]
Peru	Two surveys: 1.8 (2/110) and 1.6 (1/62)	Lettuce, mint, black mint	[138]
Venezuela	5.9% (6/102)	Lettuce	[139]
Vietnam	8.4 (24/287)	Herbs (basil, coriander sativum and coriander, Vietnamese mint, marjoram, persicaria) and lettuce	[69]

* Unclear which commodity was contaminated by *Cyclospora* spp.

**Table 2:** Summary of outbreaks associated with *C. cayetanensis* by country and year of occurrence and possible sources of exposure.

Country	Year	Disease Cases (If Laboratory Confirmed: Number of Cases)	Source of Exposure Suspected or Known	Origin/Notes	References
Australia (cruise)	2010	266 (73)	Lettuce (suspected)	Two consecutive voyages	[144]
Brazil	1999–2002	132 (16)	Not indicated	Not available	[149]
Canada (2 provinces) and USA (20 states)	1996	1465 (978)	Raspberries	Guatemala	[150,151,152]
Canada (Ontario) and USA (14 states)	1997	1012 (422)	Raspberries	Guatemala	[150,153]
Canada (Ontario)	1998	315	Raspberries	Guatemala	[99,150]
Canada (Ontario)	1999	104	Dessert berries (blackberries suspected)	Blackberries (Guatemala), strawberries (USA), frozen raspberries (Chile)	[99,150]
Canada (BC ^&^)	1999	15	Undetermined	Undetermined	[150]
Canada (BC)	2001	30 (17)	Thai basil	Imported from Thailand (supplier in USA). Not confirmed	[142]
Canada (BC)	2003	11	Cilantro	Undetermined, community	[154]
Canada (BC)	2004	17 (9)	Mango or basil (suspected)	Not confirmed, community	[154]
Canada (BC)	2004	8	Cilantro suspected	Undetermined, community	[154]
Canada (Quebec)	2005	250 (142)	Fresh basil	Mexico, workers who ate in a restaurant	[155]
Canada (Ontario)	2005	44 (16)	Basil (suspected)	Retreat. Pasta salad with basil, origin not confirmed (shipment from Peru and Costa Rica)	[154]
Canada (BC)	2006	28	Basil or garlic	Undetermined	[154]
Canada (BC)	2007	29 (14)	Organic basil	Mexico	[156]
Canada (Sarnia)	2010	210	Basil, pesto	Fundraiser event	[19]
Canada	2013	25	Leafy greens suspected	Undetermined	[157]
Canada (multiple provinces)	2015	97	Undetermined, multistate (BC: 5, Alberta: 1, Ontario: 84, Quebec: 7)	Travelers from Mexico	[115,158]
Canada (multiple provinces)	2016	87	Undetermined	BC:2, Alberta:2, Ontario 75, Quebec: 8	[158]
Canada (multiple provinces)	2017	164	Undetermined	BC (17), Ontario (143), Quebec (3) and Nova Scotia (1)	[158]
Colombia	2002	56 (31)	Salad, juices	University employees	[159]
Germany	2000	34	Salad, green leafy herbs	France, Italy, Germany	[94]
Nepal	1989	535 (55)	Drinking water suspected	Foreigners, Travelers from UK	[160]
Nepal	1989	14 (12)	Drinking water suspected	Foreigners	[161]
Nepal	1992	964 (108)	Drinking water suspected	Foreigners	[40]
Mexico	2001	97 (55/70 fecal samples analyzed)	Watercress (salad berros)	Party attendees at wedding and christening	[162]
Peru	2004	127/274 people with diarrhea, 24/77 positive by microscopy	Undetermined (salsa sauces suspected)	Recruits	[90]
Peru	2005	52 recruits (37 positive, 15 control) 20/35 positive by PCR	Undetermined (food, water)	Recruits	[89]
Poland	2013	3	Drinking water suspected	Travelers from Indonesia	[118]
Spain	2003	13 (7)	Raspberry juice suspected	Travelers from Guatemala	[113]
Sweden	2009	18 (12)	Snap peas	Guatemala	[143]
The Netherlands	2001	29 (14)	Could not investigate potential food sources	Dutch participants at a scientific meeting in Bogor, Indonesia	[121]
Turkey	2005	35	Drinking water suspected	Undetermined	[163]
Turkey	2007	505 stools (14/17 positive by PCR)	Undetermined, suspected to be related to lack of rain and use of contaminated water	Unwashed green salad suspected	[164]
United Kingdom	2015	79	Undetermined	Travelers from Mexico	[115]
USA (Illinois)	1990	21	Tap water or food	Not completely clarified	[165]
USA (Florida)	1995	38	Raspberries suspected, risk factor soil contact	(Guatemala as possible source)	[166]
USA (New York)	1995	32	Fruit suspected	Undetermined	[99]
USA (Massachusetts)	1996	57 (12)	Berries	Wedding, strawberries (California), blueberries (Florida), blackberries (Guatemala), raspberries (Guatemala/Chile)	[167]
USA (Florida)	1996	86	Raspberries (suspected)	Guatemala	[168]
USA (South Carolina)	1996	38	Raspberries	Guatemala	[169]
USA (20 states) and Canada (2 provinces)	1996	1465 (978)	Raspberries	Guatemala	[70,150,151,152,154]
USA (Florida),	1997	220 (including people in cruise ship that departed from Florida)	Mesclun	Peru or USA (If related to 12 cases from Florida above, then mesclun from Peru most probable source)	[99]
USA (14 states) and Canada (Ontario)	1997	1012 (422)	Raspberries	Guatemala	[150,153]
USA (Northern Virginia, Washington DC-Baltimore metropolitan area)	1997	341 (48)	Basil	Multiple possible sources, may be local contamination	[99]
USA (Virginia)	1997	21	Fruit plate	Undetermined	[99]
USA (Georgia)	1998	17	Probable fruit salad	Undetermined	[99]
USA (Florida)	1999	94	Berries likely	Undetermined	[99]
USA (Missouri)	1999	62	Basil in chicken pasta and tomato basil salad	Mexico or USA, two events	[170]
USA (Georgia)	2000	19	Raspberries and/or blackberries (suspected)	Suspected to be from Guatemala	[171]
USA (Pennsylvania)	2000	54	Raspberry, wedding cake	Guatemala	[141]
USA (Florida)	2001	39	Undetermined	Undetermined	[171]
USA (New York City)	2001	3	Undetermined	Undetermined	[171]
USA (Vermont)	Dec 2001-Jan 2002	22	Raspberry (likely)	Suspected to be from Chile	[171]
USA (Massachusetts)	2002	8	Undetermined	Undetermined	[171]
USA (New York)	2002	14	Undetermined	Undetermined	[171]
USA (Texas, Illinois)	2004	95 (38 in Texas, 57 in Illinois)	Undetermined	Undetermined, basil likely	[3,171]
USA (Tennessee)	2004	12	Undetermined	Undetermined	[171]
USA (Pennsylvania)	2004	96	Snow peas	Guatemala	[172]
USA (Florida)	2005	582	Basil, restaurants	Peru	[19,171]
USA (South Carolina)	2005	6	Undetermined	Undetermined	[171]
USA (Massachusetts)	2005	74	Two different outbreaks (58 and 16 cases)	Undetermined	[171]
USA (Connecticut)	2005	30	Basil suspected	Undetermined	[171]
USA (Minnesota)	2006	14	Undetermined	Undetermined	[171]
USA (New York)	2006	20	Undetermined	Undetermined	[171]
USA (Georgia)	2006	3	Undetermined	Undetermined	[171]
USA (Wisconsin)	2008	4	Sugar snap peas (likely)	Guatemala not confirmed	[171]
USA (California)	2008	45	Raspberries and/or blackberries (likely)	Undetermined	[171]
USA (District of Columbia)	2009	34	Undetermined	Undetermined	[171]
USA (Connecticut)	2009	8	Blackberries and raspberries	Undetermined	[171]
USA (Florida)	2011	12	Undetermined	Undetermined	[171]
USA (Georgia)	2011	88*	Undetermined	Undetermined	[171]
USA (Texas)	2012	16	Undetermined	Undetermined	[171]
USA (Iowa, Nebraska and neighboring states)	2013	162	Bagged salad mix	Mexico	[171]
USA (Texas)	2013	270 (38)	Cilantro	Mexico, multistate	[173]
USA (Wisconsin)	2013	8	Berry salad (suspected)	Undetermined	[171]
USA (Michigan)	2014	14	Undetermined	Undetermined	[171]
USA (Iowa, Nebraska)	2014	227 (161)	Lettuce (imported romaine lettuce)	Mexico	[146,147]
USA (Texas)	2014	304 (26)	Cilantro	Mexico	[171]
USA (South Carolina)	2014	13	Undetermined	Undetermined	[171]
USA (31 states)	2015	546 (90 cases in Georgia, Texas and Wisconsin)	Cilantro (suspected)	Mexico	[171]
USA (Texas)	2016	6 **	Carrots or green cabbage in coleslaw (suspected)	Undetermined	[171,174]
USA (40 states)	2017 (summary)	1065	Undetermined	Undetermined	[171,174]
USA (Florida)	2017	6	Berries (suspected)	Undetermined	[171]
USA (Texas)	2017	38 ***	Scallions (i.e., green onions)	Undetermined	[171]
USA (Michigan)	2017	29	Undetermined	Undetermined	[171]
USA (Tennessee)	2017	4 †††	Undetermined	Undetermined	[171]
USA (Connecticut)	2017	3	Undetermined	Undetermined	[171]
USA (Florida)	2017	3 ‡‡‡	Undetermined	Undetermined	[171]
USA (33 states)	2018 (summary)	2299 total cases (partial data indicated in a, b, c and d)	Many cases not directly linked, rest indicated in a, b, c and d cases		[175,176]
USA (4 states) ^a^	2018	250 (250)	Vegetable trays with broccoli, cauliflower, carrots and dill dip	Bought in supermarkets. It was not possible to determine if an individual component of the vegetable trays was the likely vehicle of infection	[177]
USA (15 states and New York) ^b^	2018	511 (511)	Romaine lettuce and carrot mix	Salads purchased from a fast restaurant chain	[178]
USA (2 states) ^c^	2018	8 (8)	Basil	Undetermined	[175]
USA (Midwest- 3 clusters) ^d^	2018	53 (53)	Cilantro	Mexican-style restaurants	[175]
USA (30 states) ^&^	2019	1,696 (1,696)	Basil (205)	Mexico	[175]

In bold: Outbreaks in two countries at the same time. ^&^ British Columbia. * An additional 10 probable cases associated with this outbreak according to the Centers for Disease Control and Prevention (CDC) (2017), were not included in the total. ** An additional nine suspected cases were identified in persons associated with this outbreak but were not counted in the table because of reporting issues (e.g., insufficient case data) according to CDC (2017). *** An additional three probable cases were identified in persons associated with this outbreak but were not counted in the table because of reporting issues (e.g., insufficient case data). ††† An additional two probable cases were identified in persons associated with this outbreak but were not counted in the table because of reporting issues (e.g., insufficient case data). ‡‡‡ One additional probable case was identified in a person associated with this outbreak but was not counted in the table because of a reporting issue. In 2018, a, b, c and d show cases directly linked to specific food produce. & As of August 2019.

**Table 3:** Water sources contaminated by *Cyclospora* spp. and/or *C. cayetanensis* in different countries.

Country	Type of Water	Percentage (Positive/Total Analyzed)	Reference
Cambodia	Water spinach	8.3 (3/36)	[126]
Egypt	Household water tanks in Alexandria	9.0 (9/100)	[183]
Egypt	Finished piped water, irrigation canals, shallow underground water and drain water	positive detection from 5 residential areas. Number not indicated	[72]
Egypt	Treated potable water from tanks	0.2 (2/840)	[184]
Egypt	River Nile, water works, water pumps, water tank, pond and canals	5.9 (20/336)	[185]
Ghana	Sachet drinking water	59.3 (16/27)	[186]
Guatemala	Rivers	6.7 (2/30)	[58]
Guatemala	Drinking water sources	41.7 (5/12) *	[187]
Italy	Tap water	30.0 (3/10) *	[135]
Italy	Treated wastewater	21.3 (20/94) *	[188]
Italy	Well water	6.2 (1/16) *	[188]
Malaysia	(1) Drinking water treatment plants and (2) Recreational water (man-made lake)	(1) 8.3 (2/24) and (2) 16.7 (2/12)	[189]
Nepal	Chlorinated water	presence of oocysts	[161]
Nepal	Sewage contamination	22.2 (4/18); 41.7 (5/12)	[15,71]
Nepal	Irrigation water, pond water, tap water and tube wells water	2 of 8 irrigation canals, 1 of 12 pond water samples (none in tap water or tube wells)	[76]
Peru	Wastewater	72.7 (8/11)	[182]
Spain	DWTP ^a^, WWTP ^b^, rivers	9.0 (20/223)	[190]
Tunisia	Wastewater	0.4 (1/232) *	[191]
Turkey	(1) Streams and (2) drinking water	Total: 24.6 (56/228) (1) 31.1 (56/180), (2) 0.0 (0/48)	[192]
USA	WWTP ^b^ (1) influent and (2) effluent	(1) 25.0 (6/24) (2) 12.5(3/24) *	[193]
Vietnam	Lakes and rivers	63.6 (84/132) *	[194]
Vietnam	Water samples from markets and farms	12.6 (12 /95)	[69]

* PCR methods; ^a^ Drinking water treatment plants; ^b^ Wastewater treatment plants. *Cyclospora* spp. in references [69,72,126,161,183,189,192] and *Cyclospora cayetanensis* in references [15,58,76,135,182,184,185,186,187,188,190,191,192,193,194].

## Data Availability

The present study is a review and the data used to support the findings are including within the article.
